# Impacts of PACAP 1-38 and BGP-15 on the Healing of Fasciocutaneous Groin Flaps Affected by Ischemia–Reperfusion in Rats

**DOI:** 10.3390/biomedicines13092129

**Published:** 2025-08-31

**Authors:** Anna Orsolya Flasko, Laszlo Adam Fazekas, Gergo Kincses, Adam Varga, Adam Attila Matrai, Ildiko Czirjak, Noemi Dodity, Ildiko Katalin Bacskay, Agota Peto, Dora Reglodi, Csaba Filler, Tamas Juhasz, Norbert Nemeth

**Affiliations:** 1 Department of Otorhinolaryngology and Head and Neck Surgery, Faculty of Medicine, University of Debrecen, Nagyerdei str. 98, H-4032 Debrecen, Hungary; flasko.anna.orsolya@med.unideb.hu; 2 Department of Operative Techniques and Surgical Research, Faculty of Medicine, University of Debrecen, Moricz Zsigmond str. 22, H-4032 Debrecen, Hungary; fazekas.laszlo@med.unideb.hu (L.A.F.); var-ga.adam@med.unideb.hu (A.V.); matrai.adam@med.unideb.hu (A.A.M.); cirmildo@mailbox.unideb.hu (I.C.); dodity.noemi@mailbox.unideb.hu (N.D.); 3 Department of Surgery, Faculty of Medicine, University of Debrecen, Moricz Zsigmond str. 22, H-4032 Debrecen, Hungary; kincses.gergo@med.unideb.hu; 4 Department of Pharmaceutical Technology, Faculty of Pharmacy, University of Debrecen, Ferenc Rex str. 1, H-4002 Debrecen, Hungary; bacskay.ildiko@pharm.unideb.hu (I.K.B.);; 5 HUN-REN-PTE PACAP Research Group, Department of Anatomy, Medical School, University of Pecs, Szigeti ut 12, H-7624 Pecs, Hungary; dora.reglodi@aok.pte.hu; 6 Department of Anatomy, Histology and Embriology, Faculty of Medicine, University of Debrecen, Nagyerdei krt. 98, H-4032 Debrecen, Hungaryjuhaszt@anat.med.unideb.hu (T.J.)

**Keywords:** fasciocutaneous flap, ischemia–reperfusion, PACAP, BGP-15, hemorheology, would healing, tensile strength, histology

## Abstract

**Background/Objectives**: To prevent flap failure, adequate tissue perfusion and effective regenerative processes, undisturbed wound healing are essential, among others. To improve wound healing, various locally and systematically administered pharmacons can be used. This study investigated the effect of PACAP 1-38 (pituitary adenylate cyclase activating polypeptide) and BGP-15 (a nicotinic amidoxime derivative) on the healing of epigastric fasciocutaneous flaps exposed to ischemia–reperfusion (I/R). **Methods**: Wistar rats were randomly divided into control (no substance), PACAP 1-38, and BGP-15 groups. Groin flaps were prepared bilaterally. The left flap was exposed to 120 min of ischemia prior to suturing it back. We applied wound gels containing substances. Laboratory tests (hematology, erythrocyte deformability, and aggregation) were performed before surgery on the 1st, 3rd, and 7th postoperative days. Lastly, flap skin samples were taken for histological and tensile strength measurements. **Results**: Impaired erythrocyte deformability and enhanced aggregation were found because of flap I/R. The pharmacons were able to reduce the systemic micro-rheological impairment to varying degrees. The tensile strength increased in the areas of better perfusion. **Conclusions**: The anti-inflammatory effects of PACAP 1-38 and BPG-15, as well as the impact of PACAP 1-38 on collagen and elastic fiber composition, have been demonstrated.

## 1. Introduction

Flaps are widely used in many fields for reconstruction, such as onco-surgery, traumatology, burn surgery, and plastic surgery. Flaps can be classified according to tissue composition (e.g., cutaneous, fasciocutaneous, myocutaneous, osteomusculocutaneous), location (e.g., local, regional, distant), arrangement of blood supply, configuration, and method of transfer onto the wound [1,2,3,4,5]. The choice of the ideal flap determines the success of surgery. Choosing the appropriate flap type is based on several considerations, such as localization, size and depth of the tissue defect, adjacent structures, vasculature, Langer lines of the skin, aesthetic issues, co-morbidities, and influencing factors (smoking, peripheral vascular disease, atherosclerosis, diabetes mellitus, steroid use, previous surgeries), as well as the surgeon’s skill [1,2,3,4,5,6,7].

Covering soft tissue defects, fasciocutaneous flaps (axial flaps) are widely used in reconstructive surgical procedures. These flaps consist of skin, subcutaneous tissue, and deep fascia [8,9]. The success of flap plastics is influenced by proper vascularization (e.g., pedicle, angiosome borders), prevention of thrombotic complications, reduction of the ischemic time, and promotion of wound healing [7,10,11]. For studying flap ischemia–reperfusion and regeneration, numerous animal models are known [12,13,14,15,16]. Groin flaps are easy to prepare and can be well standardized for comparative studies [16,17].

Ischemia, ischemia–reperfusion (I/R), thrombosis, and flap necrosis can be caused by several factors, including inadequate flap preparation, tissue damage, bending/torquation of the supplying vessels (pedicle), hematoma, edema, and infection [4,11,18,19]. For adequate wound healing and regenerative processes, proper tissue perfusion is essential. Tissue microcirculation is strongly determined not only by the vasculature but also by micro-rheological factors [19,20,21]. Red blood cell deformability and aggregation play an important role in tissue perfusion, and they can be worsened by inflammatory processes and I/R injuries [17,21]. Previous studies demonstrated that red blood cell deformability is impaired, and erythrocyte aggregation is enhanced in the early wound healing phase of various flaps affected by ischemia and reperfusion [17,19]. These models are suitable for further study of flap pathophysiology, factors influencing wound healing and regeneration processes, and various preventive options and agents [14,16,17].

Pituitary adenylate cyclase-activating polypeptide (PACAP) is an endogenous neuropeptide (receptors: PAC1, VPAC1, VPAC2). It stimulates the adenylyl cyclase, so it increases the 3′,5′-cyclic adenosine monophosphate (cAMP) concentration in cells. Its protective, anti-inflammatory, and anti-apoptotic effects have been revealed; furthermore, it enhances the production of extracellular matrix elements [22,23,24,25,26].

BGP-15 (C_14_H_22_N_4_O_2_·2HCl) is a nicotinic amidoxime derivative (N′-(2-hydroxy-3-(piperidin-1-yl)propoxy)-3-pyridine-carboximidamide), which was originally developed by Hungarian researchers to treat insulin resistance. Besides other effects, it also increases heat shock protein induction, reduces reactive oxygen species production, and increases membrane fluidity. It plays a protective role in inflammatory and I/R processes [27,28].

We hypothesized that I/R injury of fasciocutaneous flaps may cause micro-rheological alterations, and administration of PACAP 1-38 or BGP-15 may have a beneficial effect on wound healing. The objective of this study was to investigate the hematological, micro-rheological, biomechanical, and histological effects of inert gels containing PACAP 1-38 or BGP-15 on the regeneration of epigastric fasciocutaneous flaps exposed to I/R during the inflammatory and early granulation phase of wound healing.

## 2. Materials and Methods

### 2.1. Experimental Animals

This study was officially approved and registered by the University of Debrecen Committee of Animal Welfare and the National Food Chain Safety Office (registration number: 19/2022/UDCAW) in accordance with national (Act XXVIII of 1998 on the Protection and Humane Treatment of Animals) and EU regulations (Directive 2010/63/EU).

Twenty-one male Wistar rats (322.79 ± 17.63 g, Toxi-Coop Zrt., Budapest, Hungary) were used in the research. The rats were delivered to the department’s standard animal facility at six weeks of age and allowed to acclimatize for three weeks before the experiment began. Prior to the procedure, the animals were weighed and anesthetized with a mixture of 100 mg/bwkg i.p. ketamine (CP-ketamine hydrochloride 10%, Produlab Pharma BV, Raamsdonksveer, The Netherlands), 10 mg/bwkg i.p. xylazine hydrochloride (CP-xylazine hydrochloride, 2%; Produlab Pharma BV, Raamsdonksveer, The Netherlands), and 0.05 mg/kg Atropin s.c. (Atropinum sulfuricum 0.1%, Egis Pharmaceuticals PLC, Budapest, Hungary) [29,30]. Throughout the operation, respiratory rate and depth and color of the mucous membrane and extremities were observed. A heating pad ensured optimal body temperature during the entire surgical intervention and until the animals woke up.

### 2.2. Operative Techniques and Experimental Groups

**Figure 1:**
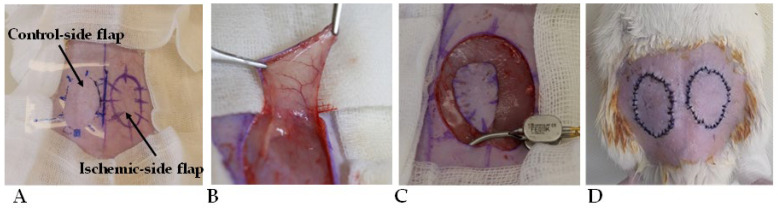
Intraoperative photos of the flap design of standard shape and size (5.48 cm^2^) (**A**), the flap preparation (**B**), the microvascular clip application to induce 120 min ischemia on the left flap (**C**), and the repositioned, sutured flaps (**D**).

In all animals, bilateral fasciocutaneous groin flaps were atraumatically prepared using microsurgical techniques. To achieve standard shape and size, a prefabricated standard-sized template was used (oval shape, surface: 5.48 cm^2^). We disinfected and isolated the surgical site, then we made the skin incision according to the mark. The pedicle of the flaps was based on the inferior superficial epigastric artery and vein. On the left side, the flap pedicle was clamped for 120 min using a microsurgical clip, while the right-side flap was left in the wound bed. After the 120 min time period, 0.3–0.3 mL hydroxypropyl-methylcellulose gel (HPMC) was applied to the wound beds. In the control group (n = 7), the gel did not contain other substances. In the PACAP group (n = 7), 0.01% PACAP 1-38 was put in the gel, and in the BGP-15 group (n = 7), 10% BGP-15 was put in the gel. The gels were prepared in the Department of Pharmaceutical Technology, Faculty of Pharmacy, University of Debrecen. Then, the flaps were sutured back to their original position, using 32 simple interrupted tension-free stitches on each flap (cutting needle, Silk, SMI, Steinerberg, Belgium) (Figure 1).

The rats postoperatively received warmed subcutaneous fluid (2 mL of 0.9% NaCl). Wound gels (according to the groups) were applied daily for 5 postoperative (p.o.) days around the suture line and on the flap surface (0.15 mL/flap/day). Postoperatively, animals were kept individually, and a plastic collar was used around the neck of the animals to prevent autophagy. To ensure an unbiased evaluation of the healing process, conventional NSAIDs were not used for postoperative pain relief. Instead, for long-lasting analgesia, Tramadol (15 mg/kg/day, s.c.) [31] was given on the first 3 p.o. days.

### 2.3. Sampling Protocol

Preoperatively (base) and on the 1st, 3rd, and 7th p.o. days, blood samples were taken from the lateral tail vein (0.4 mL/each; K3-EDTA, Vacutainer^®^, Becton Dickinson GmbH, Franklin Lakes, NJ, USA) for laboratory tests.

Besides daily wound care, photographs of the flap area were also taken on the 1st, 3rd, and 7th p.o. days. The area of the flaps was determined using ImageJ 1.40g freeware, calibrated each time using a ruler placed on the animal.

In parallel, skin temperature was measured (Ri-thermo^®^ N professional Clinical Thermometer, Rudolf Riester GmbH, Jungingen, Germany) on both flaps and on intact epigastrial skin region (middle region) before operation; during ischemia; after operation (after suturing the flaps); and on the 1st, 3rd, and 7th p.o. days.

**Figure 2:**
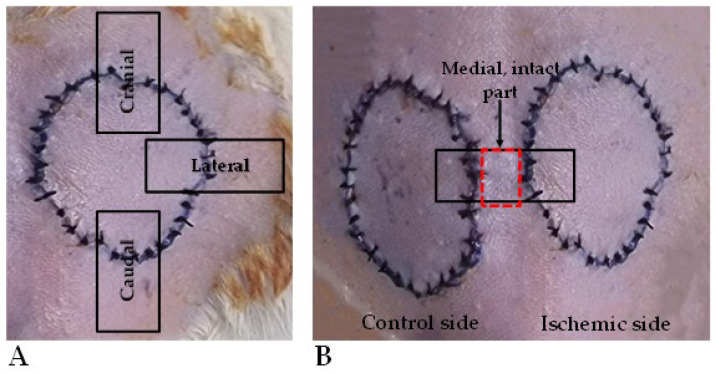
On the 7th postoperative day, during biopsy taking for the tensile-strength test, 0.5 × 3 cm stripes were excised from the cranial, lateral, and caudal regions of the flaps, including flap skin, suture line, and intact skin part (**A**). A similarly sized skin stripe was excised for histology, containing the medial flap parts and intact skin in between the flaps (**B**).

On the 7th p.o. day (at the end of the experiment), the animals were euthanized by intravenous injection of 300 mg/kg of ketamine hydrochloride 10% and 30 mg/kg of xylazine hydrochloride 2%, using a lateral tail vein catheter. Tissue samples were taken: 0.5 × 3 cm stripes were excised from the cranial, lateral, and caudal parts of the flaps, including the flap’s skin region, the suture line, and the adjacent intact skin part for tensile strength measurements (Figure 2A). Furthermore, similarly sized skin stripes were also excised from the medial parts of the flaps, together with intact skin parts for histological analyses (Figure 2B).

### 2.4. Laboratory Analysis

Qualitative and quantitative hematological variables were measured using a Sysmex K-4500 microcell counter (TOA Medical Electronics Co., Ltd., Kobe, Japan). The parameters assessed included white blood cell count (WBC [G/L]), red blood cell count (RBC [T/L]), hematocrit (Hct [%]), and platelet count (Plt [G/L]).

Red blood cell deformability was determined using a LoRRca MaxSis Osmoscan ektacytometer (RR Mechatronics International B.V., Zwaag, The Netherlands) [32]. The blood samples were subjected to shear stress, and laser diffraction techniques were used to measure red blood cell elongation. The elongation index (EI) was determined as a function of shear stress (SS, Pa; range: 0.3 to 30 Pa). The conventional deformability test was performed by carefully mixing 10 μL of sample (whole blood) with 2 mL of polyvinylpyrrolidone (PVP)–PBS solution (PVP: 360 kDa, Sigma-Aldrich Co., St. Louis, MO, USA; PVP-PBS solution viscosity = 31.6–33.6 mPas, osmolality = 290–310 mOsmol/kg, pH = 7.5). All measurements were conducted at 37 °C [33]. Comparative data from the EI–SS curves were calculated, including EI values at 3 Pa. The Lineweaver–Burk equation (1/EI = SS_1/2_/EI_max_ × 1/SS + 1/EI_max_) was used for parameterization of individual EI–SS curves, providing the maximal elongation index (EI_max_) and shear stress at half EI_max_ (SS_1/2_, Pa) [34]. Impaired red blood cell deformability is indicated by lower EI or EI_max_ and higher SS_1/2_ values [32,34].

Aggregation index values of blood samples were determined using a Myrenne MA-1 erythrocyte aggregometer (Myrenne GmbH, Roetgen, Germany). This method relies on light-transmittance photometry and requires 20 µL of blood. Following disaggregation using a controlled shearing system (shear rate: 600 s^–1^), light transmission was assessed for 5 or 10 s under static conditions (M values, shear rate: 0 s^–1^) or at a low shear rate (M1 values, shear rate: 3 s^–1^) [32]. Measurements were performed at room temperature (20–25 °C). Higher index values (M 5 s, M1 5 s, M 10 s, M1 10 s) indicate increased RBC aggregation [32,33].

### 2.5. Tensile Strength Measurement

For the tensile strength tests, skin strips with a consistent width of 5 mm were prepared on the 7th postoperative day. These samples encompassed both the intact and flap regions, along with the healed suture line at the center. We took samples from both the ischemic and non-ischemic flaps and from their cranial, caudal, and lateral parts. The samples were then placed in a tensile strength measuring device previously developed in collaboration with our department. During the test, a continuously increasing tensile force (1.95 mm/s) was applied until they ruptured. The maximum force the samples endured before breaking serves as an indicator of the tissue’s tensile strength. The force (newton) needed to cause rupture, and the degree of elongation was continuously monitored. The machine-generated stress–strain curve enabled the determination of key parameters, including maximum stress, the breaking point, and the slope of the curves [26,35].

### 2.6. Histological Analyses

The area of interest of the skin was peeled off and removed during surgery. Skin samples were fixed on dental wax, then placed in formalin for 2 days. The use of dental wax was necessary to prevent the samples from shrinking and coiling. After fixation, samples were washed 3 times in distilled water, then in a series of increasing concentrations of alcohol and embedded in paraffin. Serial sections of 7 µm thick slices were taken with microtome (Leica, Wetzlar, Germany). The samples were stained with dimethyl-methylene blue (DMMB) dissolved in water (Sigma-Aldrich, MO, USA), hematoxylin–eosin (H&E, Sigma-Aldrich, MO, USA), Orcein (Sigma-Aldrich, MO, USA), and picrosirius red staining (Sigma-Aldrich, MO, USA), according to the instructions of the manufacturer. Slices were covered with DPX (Sigma-Aldrich, MO, USA). Histological slices stained with DMMB, H&E, and Orcein were examined with a light microscope BX53 Olympus (Olympus, Tokyo, Japan) with constant camera and exposure settings. DMMB is a classic, rapid method that stains the granules within mast cells a metachromatic purple or violet color due to their high content of heparin and histamine. Mast cells appear as purple–blue granules against a lighter background. The specificity of DMMB for mast cell granules is due to its high affinity for the sulfated proteoglycans unique to mast cells.

The samples stained with picrosirius red were examined with polarization lens where the polarized light plane was rotated by λ/4 and detected with a λ/4 compensator using a BX53 Olympus microscope (Olympus, Tokyo, Japan). Photos were taken in normal light and then in polarized light with constant camera settings. Following H&E staining of the tissue samples, the morphological differences between the groups were compared, and the thickness of the epidermis was measured (ImageJ 1.40g freeware). After DMMB staining, mast cells were counted in the area of interest, and total cell number was given. Furthermore, in Picrosirius red staining, the pixel intensity of green (thin) and red (thick) collagen fibers was also measured by ImageJ 1.40g freeware, and semiquantitative data were given and normalized to the data of intact skin.

### 2.7. Statistical Methods

The Mead’s resource equation method [36] was used to determine the sample size (as number of animals per group). For statistical analyses, SigmaStat Software 3.1.1.0. (Sys-tat Software Inc., San Jose, CA, USA) was used.

Data are presented as means ± standard deviation (S.D.). For inter-group comparison, *t*-test or the Mann–Whitney rank-sum test was used; for intra-group comparison, one-way ANOVA or Kruskal–Wallis’s test was used (depending on the result of data distribution normality test). A *p*-value of <0.05 was considered statistically significant.

## 3. Results

### 3.1. Macroscopic Evaluation

During the postoperative observation period, we did not see significant thrombotic complications or flap necrosis. The surface of the flaps significantly decreased by the 1st p.o. day in all groups. The reduction of flap surface area continued during the observation period, especially in the ischemic flaps. There were no significant differences between groups, but the largest decrease on 7th p.o. day, compared to their own first day value, was on the ischemic side (control: 24.27%; PACAP: 32.6%; BGP-15: 30.6%), while a smaller decrease was observed on the control side (control: 25.2%; PACAP: 15%; BGP-15: 13.4%). For the control group, both sides showed similar decreases, while for the treated groups, the non-ischemic side showed a smaller decrease, and even an increased value was observed on the 7th p.o. day in the PACAP group (Table 1).

### 3.2. Skin Temperature

The temperature of the skin surface, as expected, significantly decreased during the ischemic period on both sides, as all the flaps were moved from their original anatomical position. The temperature of the ischemic flaps was slightly lower compared to the non-ischemic (control-side) flaps. After repositioning and re-suturing the flaps, the temperature normalized. There was no significant difference between the non-ischemic and ischemic flaps during the postoperative days; however, in ischemic flaps, the values were slightly lower (Table 2).

### 3.3. Hematological Parameters

White blood cell count decreased by the 1st p.o. day, predominantly in the control group. By the 7th day, values increased close to the base data. In the BGP-15 group, we found lower values on the 3rd p.o. day compared to the other groups. Red blood cell count showed a slight decrease over the observation period, with an initial hemoconcentration by the 1st day in PACAP and BGP-15 groups. The hematocrit values changed accordingly. Platelet count continuously increased, showing the highest values in the BGP-15 group (Table 3).

### 3.4. Micro-Rheological Parameters

**Figure 3:**
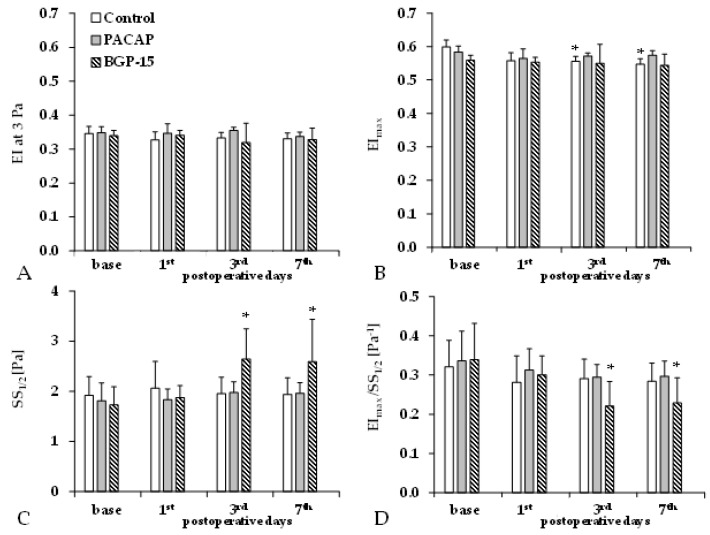
Changes in red blood cell deformability parameters in the control, PACAP, and BGP-15 groups before operation (base) and on the 1st, 3^rd^, and 7th postoperative days: elongation index at 3 Pa (EI at 3 Pa, **A**), maximal elongation index (EI_max_, **B**), shear stress at half EI_max_ (SS_1/2_, **C**), and their ratio (**D**). Means ± S.D.; * *p* < 0.05 vs. base.

Elongation index (EI) decreases as red blood cell deformability becomes impaired. We observed a slight decrease in EI values at a shear stress of 3 Pa and maximal elongation index values (EI_max_) during the p.o. days in all groups. The shear stress at half EI_max_ (SS_1/2_) only significantly increased in the BGP-15 group (Figure 3).

**Figure 4:**
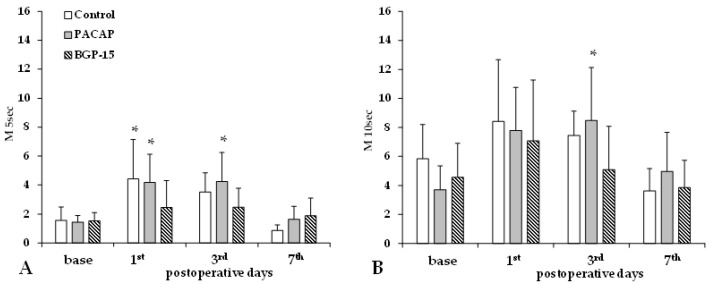
Red blood cell aggregation index values M 5 s (**A**) and M 10 s (**B**) in the control, PACAP, and BGP-15 groups before operation (base) and on the 1st, 3rd, and 7th postoperative days. Means ± S.D.; * *p* < 0.05 vs. base.

Aggregation index values (M 5 s, M 10 s) significantly increased by the 1st and 3rd p.o. days in the control and PACAP groups, while the increase in the BGP-15 group was much smaller and non-significant (Figure 4).

### 3.5. Tensile Strength

The tensile strength of the flaps (suture line) was reduced by more than 70% compared to intact skin without follow-up (intact skin strip: 17.01 ± 3.65 N; skin strip torn after suturing: 10.68 ± 2.31 N). Suture lines in the lateral skin stripes were weaker compared to the vertically oriented ones. Compared to the control, the PACAP group showed a significant (*p* = 0.0025) decrease in the tensile strength of the ischemic side. The slope of the curves was significantly increased in the PACAP-treated control lobe for the skin strip with horizontal (lateral) orientation (*p* = 0.0116 vs. BGP-15), whereas the ischemic lateral lobe was significantly decreased (*p* = 0.0077 vs. control; *p* = 0.0241 vs. BGP-15). In the BGP-15 group, only the slope of the caudal vertical skin part showed a significant (*p* = 0.0171) decrease compared to the controls (Table 4).

### 3.6. Histological Changes

**Figure 5:**
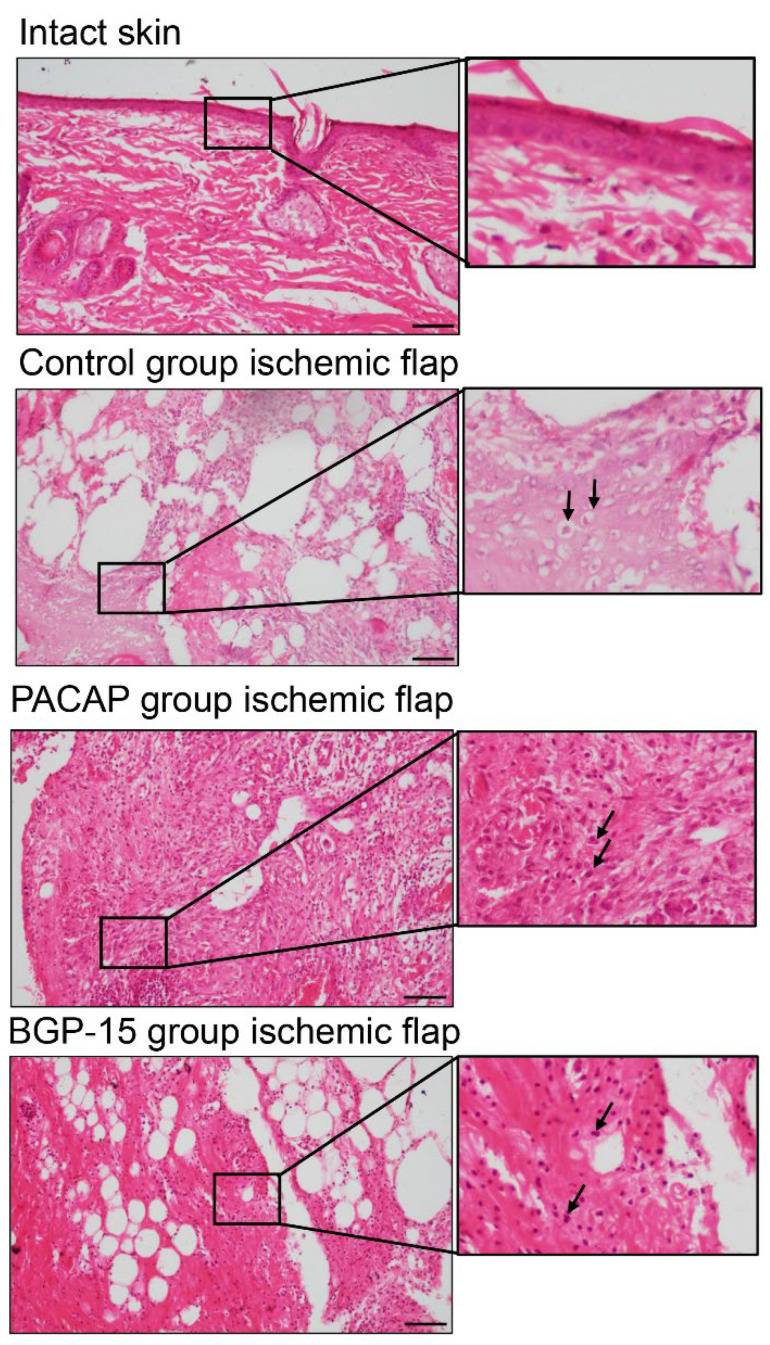
Representative H&E-stained histological pictures of intact skin and ischemic flaps of the control, PACAP, and BGP-15 groups. Sampling: 7th postoperative day. Arrows pointing at necrotic cells, fibroblasts, and macrophages. Original magnification: 20×. Scale bar: 100 µm.

H&E staining revealed sterile acute inflammatory processes with dominant lymphocyte infiltration. Around the sutures, unnucleated necrotic foam cells were seen (labeled by arrows), mostly in the ischemic flaps. Fibrocytes and fibroblasts were seen in the granulation tissue, consistent with physiological wound healing. In the ischemic-side flaps, tissue degradation was detected. In the PACAP group, a higher number of macrophages were observed with a large number of fibroblasts (labeled by arrows). Tissue degradation was not identified, but strong tissue remodeling was in progress. In the BGP-15 group, macrophages and fibroblasts (labeled by arrows) were observed in ischemic flaps. Although the tissue remodeling was not strongly demonstrated, tissue degradation was not identifiable in these groups (Figure 5).

Epidermis thickness above the granulation tissue at the sutures markedly increased in the flaps, especially in ischemic ones. The increase was smaller in the PACAP group and larger in the BGP-15 group (Table 5).

**Figure 6:**
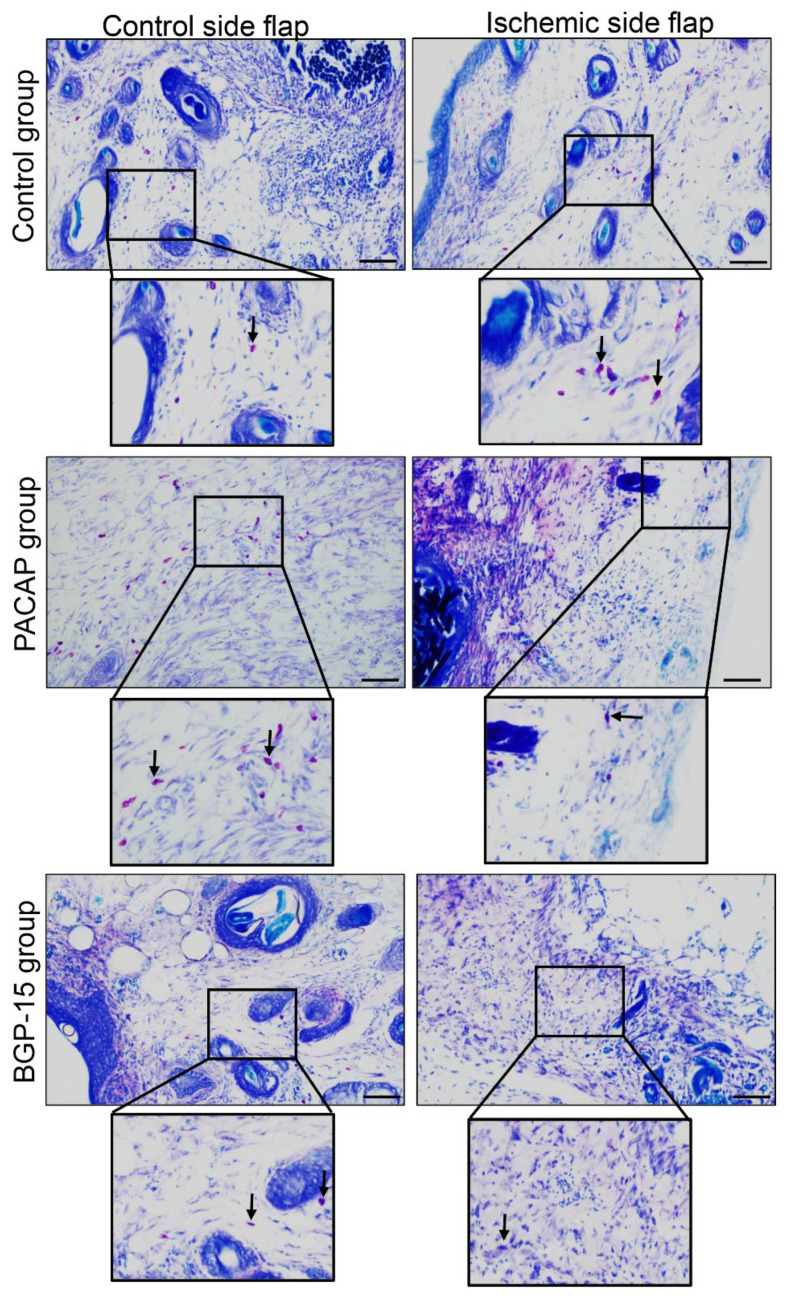
Representative dimethyl-methylene blue-stained histological slides of control-side and ischemic-side flaps of the control, PACAP, and BGP-15 groups. Sampling: 7th postoperative day. Original magnification: 20×. Scale bar: 100 µm. Inserts show magenta color mast cells labeled by arrows.

The number of mastocytes decreased in the ischemic flaps of the PACAP group compared to the control. On the other hand, PACAP decreased the number of mast cells in the control-side flap group as well. A smaller number of mast cells was demonstrated in the ischemic flaps of the BGP-15 group. On the contrary, a higher number of mast cells was detectable in the control-side flap BGP-15’s group than in the control group (Figure 6, Table 5).

**Figure 7:**
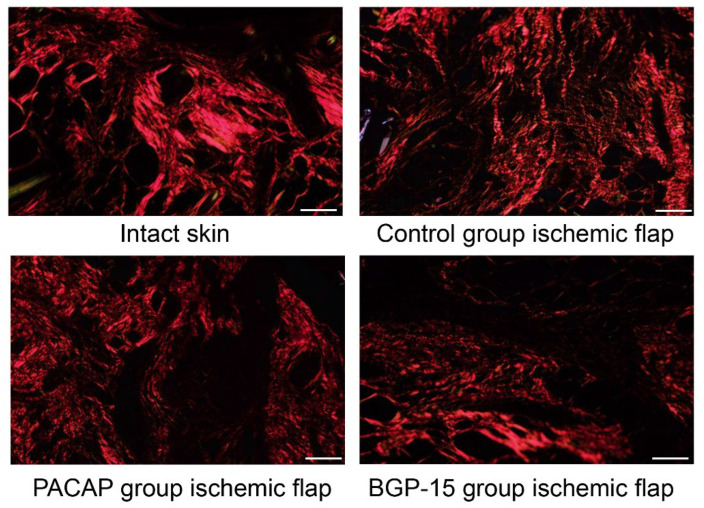
Representative picrosirius-stained histological pictures (polarized light, rotation: λ/4) of intact skin and ischemic flaps of the control, PACAP, and BGP-15 groups. Sampling: 7th postoperative day. Original magnification: 20x. Scale bar: 100 µm.

Picrosirius staining visualizes thick collagen fibers without specificity in a shiny red color, and thin collagen fibers can be identified in a green color in polarized light (rotated the light plane by λ/4). In intact skin, the dermis exhibited a dense network of thick collagen fibers consistent with physiologically dense, irregular connective tissue. In the ischemic flap group, a more diffuse and thinner fiber network was observed, corresponding to the loosening of the dense connective tissue during wound healing. Treatments with PACAP and BGP-15 reorganized the diffuse orientation of collagen fibers and resulted in the appearance of thicker, dense connective tissue characteristic of the dermis. In the BGP-15 group, thinner green color fibers were also demonstrated (Figure 7, Table 6).

**Figure 8:**
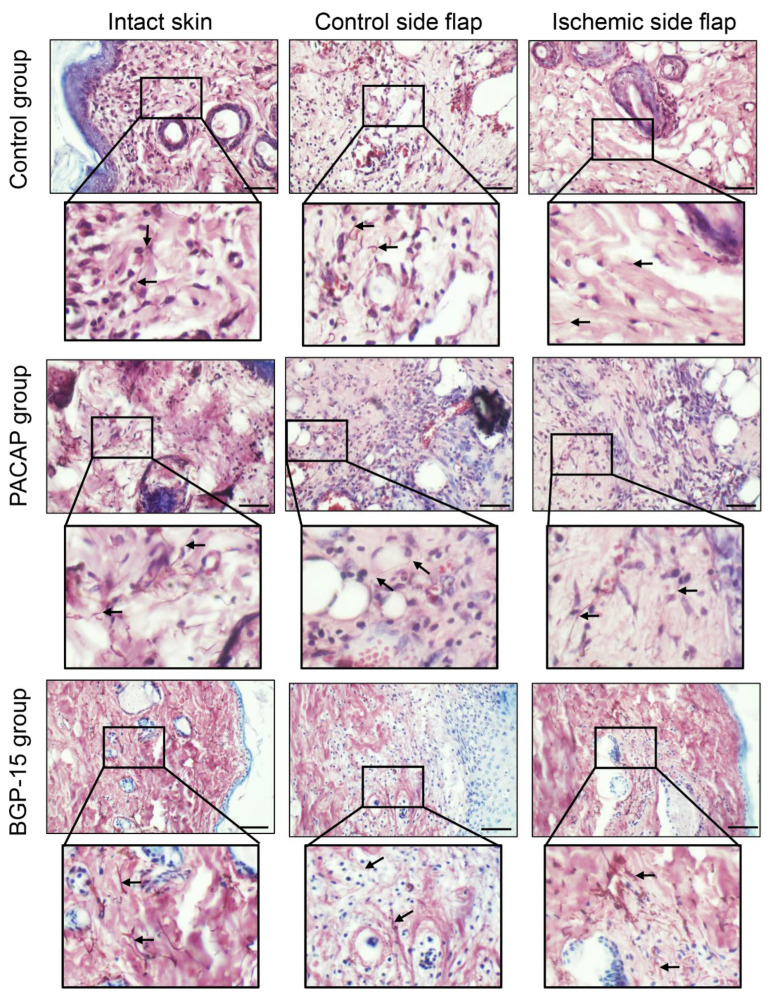
Representative orcein-stained histological photos of control-side and ischemic-side flaps of the control, PACAP, and BGP-15 groups. Sampling: 7th postoperative day. Original magnification: 40x. Scale bar: 50 µm. Brown color shows elastic fibers.

Orcein staining demonstrates elastic fibers in a brown color. In normal intact skin, within the dermis, brown-stained fibers can be observed in the physiologically dense, irregular connective tissue. These fibers varied in length and overlapped with collagen fibers, thereby ensuring the skin’s elasticity. On the control side, the amount of these orcein-stained elements was less visible in the PACAP-treated group. In the presence of the neuropeptide, a markedly higher presence of thinner elastic fibers was observed in the developing scar tissue compared to the control ischemic side flap group. Following BGP-15 treatment, the number of orcein-stained fibers on the control flap side was similar to that of the control group, while on the ischemic side, they were elevated, and thicker elastic fibers were demonstrated (Figure 8).

## 4. Discussion

Ischemia of flaps results in the cessation of tissue perfusion, hypoxia, and a decrease in flap temperature, causing cellular damage. Depending on the magnitude and duration of ischemia, it may lead to necrosis. During reperfusion, inflammatory processes, free radical reactions (oxidative stress), and metabolite accumulation lead to additive damage, including micro-rheological and microcirculatory disturbances that cause further deterioration in tissue perfusion [20,21]. We found micro-rheological impairment in all groups, however, to varying degrees. Acute phase reactions caused by the surgery itself, the tissue preparation, and the healing of more than 16 cm suture lines on each animal can explain the alterations of leukocyte count, transient hemoconcentration, rise in platelet count, as well as the increase in red blood cell aggregation index and the decrease in red blood cell deformability [17,20,21]. Previous studies on flap ischemia–reperfusion showed similar results [17].

Related to micro-rheological effects in flap I/R, the PACAP 1-38 and the BGP-15 have not been investigated yet. We mixed the agent with hydroxypropyl methylcellulose gel, which is a hydrophilic carrier with good biocompatibility, film-forming ability, and good biological degradation characteristics.

The results we found are related to the systemic effects of these agents. However, the dominant impact was expected locally in the healing wounds. Therefore, we focused on the early phase of wound healing, investigating the effect of locally administered agents (in gel). We were also interested in biomechanical and histomorphological alterations.

Multiple factors affect the tensile strength and elongation of the samples: the orientation of sampling (since skin is anisotropic, its mechanical properties vary with direction), moisture levels, and temperature [37]. Sample thickness also plays a critical role (thicker samples generally exhibit higher tensile strength), as does the organization of collagen fibers within the skin (their alignment and density influence the tissue’s resistance). Additionally, oxidative stress, such as tissue damage or inflammation, can diminish the flap’s tensile strength [18].

The findings from the tensile strength tests provide insights into the mechanical properties of the flaps, aiding in the evaluation of their load-bearing capacity, resilience to postoperative stresses, and the refinement of sample selection for specific surgical approaches. This testing method offers a reliable way to gather accurate and reproducible data on the mechanical behavior of skin flaps, which is essential for both clinical and research purposes.

The epidermis is the outermost layer of the skin, which interacts with the external environment. It provides protection against mechanical, chemical, microbial, and immunological damage and participates in thermoregulation, UV protection, and sensory perception. Thus, measuring its thickness is a quantitative method to assess its regeneration after injury. The epidermis thickness was measured above the granulation tissue and not in the middle of the flap, but on the immediate two edges of the suture line. Thickening of the epidermis may reflect the processes in the deeper layer of the tissue. Increased levels of fibroblast growth factors can directly stimulate the basal layer of the epidermis. Histology showed the thickening of the stratum polygonale. PACAP 1-38 was able to reduce tissue degradation, the degree of inflammation, and the extension of cell damage. On the control side (non-ischemic flaps), the epidermis thickness was almost the same as in the intact skin area. The BGP-15 could not reduce I/R damage, but a beneficial effect could be seen in the control group, as anti-inflammation was visible.

The examination of mast cells was justified as they play a crucial role in wound healing through the release of inflammatory factors. These factors include histamine, pro-teases, prostanoids, leukotrienes, heparin, and numerous cytokines (TNFα, IL-4, IL-5, IL-6, IL-10), chemokines, and growth factors [38,39,40,41,42]. Mastocytes may directly influence the wound healing process, as the inflammatory factors released by them are responsible for non-neural vascular processes (vessel dilatation, better perfusion, and leukocytes). By PACAP 1-38 administration, their number was reduced predominantly in the ischemic-side flap. BGP-15 had a weaker effect on modulating the mastocyte number. PACAP 1-38 also modified the migration of macrophages. However, further studies are needed with longer follow-up periods and with different dosages and routes of administration to optimize the effects of PACAP 1-38 and BGP-15 on wound healing processes, including flap regeneration.

Limitations of this study include the relatively low case number, the short follow-up period, the single route of pharmacon administration (without investigating, e.g., co-administration), the anesthesia protocol used, as well as the different wound healing dynamics of rats compared to humans. Additionally, the interspecies differences of micro-rheological parameters and their comparability have to be also taken into consideration as a limitation [21]. In this study, we could not use more sophisticated methods for ultrastructural morphological analysis; however, e.g., electron microscopy would reveal better cell damage/cell death [43,44,45,46]. We did not investigate more factors that could influence wound healing, such as metabolic conditions and metabolic disorders, which are also well-known in clinical practice [47,48,49,50].

Besides its limitations, this well-standardizable rat model seems to be useful for studying flap physiology and pathophysiology. In future studies, we wish to extend the palette of investigative methods with relevant microcirculatory [51,52] and ultrastructural [43,45,46] approaches to include metabolic reprogramming (glucose, lipids, amino acids) [49] and further therapeutic approaches, such as platelet-rich plasma for its angiogenic and anti-inflammatory effects [53].

## 5. Conclusions

The micro-rheological damage caused by ischemia–reperfusion of the fasciocutaneous flap was manifested in impaired red blood cell deformability and enhanced red blood cell aggregation. The pharmacons we used (PACAP 1-38, BGP-15) were able to reduce the systemic micro-rheological impairment caused by the ischemia–reperfusion process to various degrees.

In flaps exposed to ischemia–reperfusion, the tensile strength has been increased as a result of fibrotic matrix accumulation in the areas with better perfusion.

The applied pharmacons were able to mitigate the damaging effects on the flap tissue to a different extent. The results of anti-inflammatory (PACAP 1-38, BPG-15) and fiber composition effects (PACAP 1-38) are encouraging for further investigations with a longer follow-up period.

## Figures and Tables

**Table 1:** Decrease in the control-side and ischemic-side flaps’ surface area by the 1st, 3rd, and 7th postoperative (p.o.) days in the control, PACAP, and BGP-15 groups.

Variable	Group	1st p.o. Day	3rd p.o. Day	7th p.o. Day
Flap surface area [cm^2^] on control side	Control	3.89 ± 0.17 *	3.30 ± 0.21 *	2.91 ± 0.31 *
	PACAP	4.00 ± 0.10 *	3.22 ± 0.50 *	3.40 ± 0.44 *
	BGP-15	3.72 ± 0.32 *	3.29 ± 0.20 *	3.22 ± 0.12 *
Flap surface area [cm^2^] on ischemic side	Control	3.75 ± 0.30 *#	3.18 ± 0.07 *#	2.84 ± 0.31 *
	PACAP	3.90 ± 0.11 *#	3.15 ± 0.14 *#	2.63 ± 0.36 *
	BGP-15	3.69 ± 0.18 *	3.26 ± 0.13 *	2.56 ± 0.47 *

Means ± S.D.; * *p* < 0.05 vs. base (standardized flap surface: 5.48 cm^2^); # vs. 7th p.o. day.

**Table 2:** Changes in skin temperature [C°] on intact skin parts and on the control-side and ischemic-side flaps before flap preparation; during the ischemic period; after the operation (sutured flaps); and on the 1st, 3rd, and 7th postoperative (p.o.) days in the control, PACAP, and BGP-15 groups.

Region	Group	Before Operation	During Ischemia	After Operation	1st p.o. Day	3rd p.o. Day	7th p.o. Day
Intact skin	Control	32.61 ± 3.03	31.70 ± 2.18	32.49 ± 2.09	29.58 ± 1.63	32.41 ± 3.05	32.70 ± 1.13
	PACAP	32.44 ± 1.68	32.43 ± 1.79	32.81 ± 1.88	31.64 ± 0.95	31.67 ± 0.58	31.38 ± 1.44
	BGP-15	31.36 ± 1.05	32.00 ± 0.63	31.03 ± 1.28	33.74 ± 3.39	33.49 ± 2.90	33.62 ± 2.43
Control-side flap	Control	32.34 ± 2.89	30.14 ± 3.06 *	31.84 ± 2.57	29.25 ± 1.98	32.13 ± 3.36	32.30 ± 1.05
	PACAP	31.51 ± 1.22	29.26 ± 0.69 *	30.87 ± 1.54	30.14 ± 1.33	30.87 ± 1.13	32.22 ± 2.76
	BGP-15	31.43 ± 0.56	29.81 ± 1.27 *	30.41 ± 1.34	33.29 ± 3.47	32.31 ± 3.47	32.06 ± 1.67
Ischemic-side flap	Control	32.35 ± 2.81	30.16 ± 3.28 *	31.78 ± 2.57	29.38 ± 1.92	32.31 ± 3.20	31.71 ± 0.59
	PACAP	31.39 ± 1.05	28.96 ± 0.84 *	30.30 ± 1.30	30.07 ± 1.28	30.43 ± 1.39	31.23 ± 1.46
	BGP-15	31.23 ± 0.58	29.71 ± 1.73 *	30.16 ± 1.40	33.06 ± 3.67	32.25 ± 3.43	31.99 ± 1.56

Means ± S.D.; * *p* < 0.05 vs. before operation and vs. intact skin.

**Table 3:** Alterations of white blood cell count, red blood cell count, hematocrit, and platelet count in the control, PACAP, and BGP-15 groups before operation (base) and on the 1st, 3rd, and 7th postoperative days.

Variable	Group	Base	1st p.o. day	3rd p.o. day	7th p.o. day
White blood cell count [10^9^/L]	Control	8.59 ± 1.94	5.62 ± 3.11	8.01 ± 1.21	8.98 ± 3.71
	PACAP	9.84 ± 2.54	8.65 ± 3.13	8.51 ± 1.79	9.26 ± 1.74
	BGP-15	8.00 ± 1.91	7.9 ± 2.47	6.56 ± 0.97	8.96 ± 1.63
Red blood cell count [10^12^/L]	Control	7.23 ± 0.62	7.28 ± 0.58	7.15 ± 0.24	6.68 ± 0.71
	PACAP	7.36 ± 0.39	7.44 ± 0.94	7 ± 0.47	7.16 ± 0.39
	BGP-15	7.11 ± 10.68	7.41 ± 0.27	7.07 ± 0.31	7.08 ± 0.66
Hematocrit [%]	Control	41.71 ± 3.51	41.41 ± 3.13	40.44 ± 1.65	37.05 ± 3.83
	PACAP	42.36 ± 2.02	42.49 ± 1.25	39.99 ± 2.65	40.74 ± 2.11
	BGP-15	40.75 ± 3.84	41.63 ± 1.83	39.75 ± 2.24	39.73 ± 3.23
Platelet count [10^9^/L]	Control	689.93 ± 83.54	654.56 ± 65.52	730.43 ± 83.61	735.6 ± 107.25
	PACAP	780.22 ± 57.17	728.67 ± 112.33	640.75 ± 91.77	816.07 ± 99.17
	BGP-15	757.5 ± 94.59	672.2 ± 48.11	660.1 ± 105.81	922.3 ± 80.25 *

Means ± S.D.; * *p* < 0.05 vs. base.

**Table 4:** Alterations of tensile strength and the slope of the force–elongation curves in the cranial, lateral, and caudal flap parts of control-side and ischemic-side flaps of the control, PACAP, and BGP-15 groups.

Variable	Flap	Flap Region	Group		
			Control	PACAP	BGP-15
Tensile strength [N]	Control-side flap	Cranial	2.51 ± 0.82	2.09 ± 0.53	2.72 ± 1.09
		Lateral	1.98 ± 0.73	2.06 ± 0.47	2.14 ± 0.62
		Caudal	3.14 ± 1.09	2.00 ± 0.34	2.41 ± 0.82
	Ischemic-side flap	Cranial	3.19 ± 0.88	2.72 ± 1.28	3.02 ± 0.83
		Lateral	1.98 ± 0.34	1.04 ± 0.17 *	1.95 ± 0.69
		Caudal	2.39 ± 0.72	2.61 ± 0.75	2.83 ± 1.04
Slope of curve	Control-side flap	Cranial	0.018 ± 0.009	0.016 ± 0.003	0.013 ± 0.003
		Lateral	0.015 ± 0.008	0.023 ± 0.002 #	0.015 ± 0.004
		Caudal	0.021 ± 0.007	0.015 ± 0.002	0.009 ± 0.003 *
	Ischemic-side flap	Cranial	0.024 ± 0.004	0.0153 ± 0.004 *	0.014 ± 0.008
		Lateral	0.03 ± 0.004	0.016 ± 0.005 *#	0.043 ± 0.008
		Caudal	0.014 ± 0.005	0.02 ± 0.005	0.02 ± 0.011

Means ± S.D.; * *p* < 0.05 vs. control group; # vs. BGP-15 group.

**Table 5:** Changes in epidermis thickness and the number of mastocytes in the intact skin part and in the control-side and ischemic-side flaps of the control, PACAP, and BGP-15 groups.

Region/Flap	Group	Epidermis Thickness [μm]	Number of Mastocytes per 0.5 mm^2^
Intact skin	Control	182.00 ± 79.57	12.4 ± 4.9
	PACAP	157.86 ± 59.38	11.0 ± 4.6
	BGP-15	155.81 ± 60.65	9.6 ± 6.5
Control-side flap	Control	397.04 ± 225.41	9.8 ± 5.1
	PACAP	166.36 ± 57.74	7.75 ± 3.2 *
	BGP-15	285.21 ± 132.63	13.6 ± 1.2 *
Ischemic-side flap	Control	352.39 ± 232.32	2.2 ± 1.3 *
	PACAP	271.73 ± 121.03	1.75 ± 0.9 *#
	BGP-15	418.48 ± 228.58	1.6 ± 1.26 *#

Means ± S.D.; * *p* < 0.05 vs. intact skin; # vs. control side.

**Table 6:** Numerical analysis of the light intensity of picrosirius-stained histological samples of intact skin parts and ischemic flaps of the control, PACAP, and BGP-15 groups.

Sample	Red Light Intensity [PN]	Green Light Intensity [PN]
Intact skin	15,830.95 ± 5516.57	1542.27 ± 581.07
Control group, ischemic flaps	8979.15 ± 2468.27 *	4058.43 ± 1166.42 *
PACAP group, ischemic flaps	15,946.46 ± 2195.89	2735.57 ± 1492.07
BGP-15 group, ischemic flaps	12,867.00 ± 7271.77	3222.67 ± 1494.55

Means ± S.D.; * *p* < 0.05 vs. intact skin.

## Data Availability

The data presented in this study are available on request from the corresponding author. The data are not publicly available due to ethical constraints.
